# Identifying the risk of Kawasaki disease based solely on routine blood test features through novel construction of machine learning models

**DOI:** 10.1016/j.csbj.2025.06.037

**Published:** 2025-06-25

**Authors:** Tzu-Hsien Yang, Ying-Hsien Huang, Yuan-Han Lee, Jie-Nan Lai, Kuang-Den Chen, Mindy Ming-Huey Guo, Yan Pan, Chun-Yu Chen, Wei-Sheng Wu, Ho-Chang Kuo

**Affiliations:** a Department of Biomedical Engineering, National Cheng Kung University, University Road, 701 Tainan, Taiwan; b Medical Device Innovation Center, National Cheng Kung University, University Road, 701 Tainan, Taiwan; c Kawasaki Disease Center, Kaohsiung Chang Gung Memorial Hospital and Chang Gung University College of Medicine, Kaohsiung, 83301, Taiwan; d Department of Pediatrics, Kaohsiung Chang Gung Memorial Hospital and Chang Gung University College of Medicine, Kaohsiung, 83301, Taiwan; e Department of Electrical Engineering, National Cheng Kung University, University Road, 701 Tainan, Taiwan; f Institute for Translational Research in Biomedicine, Liver Transplantation Program and Department of Surgery, Kaohsiung Chang Gung Memorial Hospital and Chang Gung University College of Medicine, Kaohsiung, 833401, Taiwan; g Department of Pediatrics, The First Affiliated Hospital of Yangtze University, Jingzhou, 434023, Hubei Province, China; h Department of Pediatrics, Chi Mei Medical Center, Zhonghua Rd., Tainan, 71004, Taiwan; i Chia Nan University of Pharmacy and Science, Erren Rd., Tainan, 717301, Taiwan; j Department of Respiratory Therapy, Kaohsiung Chang Gung Memorial Hospital, Kaohsiung, 83301, Taiwan

**Keywords:** Kawasaki disease, Kawasaki disease diagnosis, Eosinophil, Alanine aminotransferase, C-reactive protein

## Abstract

Kawasaki disease (KD) is a leading cause of acquired coronary vasculitis in children and remains a critical diagnostic challenge among febrile pediatric patients. To support frontline pediatricians with a more objective diagnostic tool, we developed and implemented KDpredictor, a machine learning-based model for KD risk identification. KDpredictor leverages only the routine blood test features, including complete blood count with differential count, C-reactive protein, and alanine aminotransferase. It also takes the lead in using age-calibrated eosinophil, platelet, and hemoglobin results. Trained using the light gradient boosting machine algorithm on clinical data from 1,927 KD cases and 45,274 febrile controls, KDpredictor achieved strong performance metrics (auROC: 95.7%, auPRC: 72.4%, recall: 0.89) on a reserved test set, outperforming previous models by at least 3% in auROC and 39.3% in auPRC. Additional explainable AI analyses revealed that several top predictive features in KDpredictor are consistent with prior clinical findings. We also evaluated KDpredictor on three independent cohorts collected in East Asia (Taiwan and China) during the COVID-19 period. KDpredictor achieves recall values of 90.9%, 83.7%, and 91.7% on KD samples identified in three independent medical centers, respectively, indicating its applicability across independent clinical settings. In summary, KDpredictor demonstrates robust generalizability in KD risk identification across populations by using only standard blood samples independent of clinical symptoms. KDpredictor is freely available at https://cosbi.ee.ncku.edu.tw/KD_under7/.

## 1 Introduction

Fever is the most common reason young children seek medical care in pediatric emergency departments [1]. While many febrile episodes are due to self-limited viral infections, more serious causes include bacterial infections, hematologic or oncologic emergencies, and rheumatic diseases [2], [3]. Among these, Kawasaki Disease (KD) is of particular concern, especially in Asian countries such as Japan, Korea, China, and Taiwan [4], [5], [6], due to its potential to cause acquired coronary vasculitis in children under five years of age [7]. The most severe complications of KD include coronary artery lesions (CAL) and acquired heart disease [8], thus drawing growing attention to its diagnosis and management [4], [6]. Timely diagnosis and prompt treatment for KD are critical to prevent the main complications of CAL [9], making early and accurate identification of KD in febrile children essential in pediatric clinical practice [10].

The diagnosis of KD currently relies on the patient's clinical presentation [8]. Classic KD is defined by a prolonged fever lasting more than five days, accompanied by at least four of the following five features: erythema of the oral mucosa (strawberry tongue or fissured lips), bilateral non-suppurative bulbar conjunctivitis, cervical lymphadenopathy, edema or erythema of the hands or feet with following peeling, and a polymorphous skin rash [8]. However, these KD clinical signs are similar to other common febrile illnesses in children [11], [12], raising a diagnostic challenge for frontline clinicians in pediatric emergency settings. Worse still, 20%-30% of KD patients show incomplete or atypical symptoms that do not fully meet the classical diagnostic criteria, making timely recognition particularly difficult for less experienced pediatricians [8], [13]. Incomplete KD has become increasingly prevalent, now accounting for over 20% of acute KD cases [14], with a disproportionately high occurrence in infants under six months of age [15]. Given these diagnostic challenges, there is a critical need for a simple and reliable tool to distinguish KD from other febrile conditions in young children.

Several models have been developed to assist in the identification of KD, primarily using two types of input features: clinical signs and laboratory blood test results. Xu et al. [16] and Lee et al. [17] constructed deep convolutional neural network models using clinical symptom images for KD identification, while Lam et al. [18] and Ling et al. [11], [19] combined clinical symptom images with 12 to 17 laboratory features to enhance the diagnostic performance on KD. More recently, Zhang et al. [20] and Zhong et al. [21] proposed models based on gene expression profiling to classify KD. Although some of these approaches have achieved reasonable recalls (i.e., sensitivity), many rely heavily on the accurate interpretation of subjective clinical features. This dependency presents a challenge as KD symptoms often overlap with those of other febrile illnesses, making the recognition results less intuitive and potentially difficult to interpret, particularly for less experienced pediatricians. As a result, the applicability of such tools in frontline settings remains limited. To date, no KD identification tool has been developed that relies solely on routine blood tests, which would provide a more objective and accessible solution for first-line clinicians.

Our previous work demonstrated that KD could be related with biological parameters from routine blood tests through a logistic regression model, with a moderate recall of 82.4% [22]. Building on that foundation, this present study aimed to expand the patient population and enhance KD risk estimation by developing a more advanced analytical strategy. To ensure full reliance on objective and quantifiable patient data, we developed KDpredictor, a machine-learning tool based on the light gradient boosting machine (LightGBM) algorithm. KDpredictor utilizes features derived solely from routine blood tests of febrile children without incorporating any clinical diagnostic criteria for KD. We showed that KDpredictor demonstrates good performance and generalizes well (test auROC, auPRC, recall values of 99.7%, 96.8%, and 0.97, respectively). Notably, when existing works were reduced to incorporate only their selected laboratory features, KDpredictor achieved an improvement of at least 30.5% in auPRC (96.8% vs. 66.3%) and 10% in recall (97% vs. 87%) over existing tools. To gain insights into the model's decision-making process, we also analyzed the key features contributing to KD risk estimation in KDpredictor. Several of the top-ranking features utilized by KDpredictor were consistent with findings from previous clinical studies, reinforcing its biological relevance and interpretability. Additional verification on independent KD cohorts reveals the robustness of KDpredictor across different clinical settings. We believe that the high accuracy, generalizability, and interpretability of KDpredictor make it a valuable tool for clinical application and future KD research. The tool is freely accessible at https://cosbi.ee.ncku.edu.tw/KD_under7/.

## 2 Datasets and methods

### 2.1 The study population

This study is a retrospective case-control analysis conducted across the four major branch hospitals of the Chang Gung Medical Foundation in Taiwan. Collectively comprising over 9,000 inpatient beds, these hospitals include two tertiary medical centers located in Linkou and Kaohsiung, and two regional hospitals in Keelung and Chiayi. Patient data were collected from January 2010 to December 2019. All enrolled KD patients met the diagnostic criteria defined by the American Heart Association [8]. Given the high incidence of KD in children under seven years of age (>99% are under seven years of age in our database), the study was restricted to this age group. In some of the collected data, missing values occurred. To ensure a comprehensive analysis of blood test features, only patients with complete laboratory data, i.e., no missing values, were included in the development of the full (feature) KDpredictor model. Among the total 2,861 KD and 82,735 febrile control (FC) cases initially collected, 1,785 KD patients and 45,274 non-KD febrile control patients had complete blood test records and were retained for model construction. All participants were of Han Chinese ethnicity from Taiwan. Both KD and the non-KD febrile controls (FC) were recruited from pediatric emergency departments of the same hospitals during the same study period. Additionally, to assess generalizability of the developed model during the COVID-19 era, 142 KD cases diagnosed from 2020 onward were included as an independent test set. The study protocol was reviewed and approved by the Institutional Review Board of Chang Gung Medical Foundation (IRB numbers: 202100084B0, 202102568B0, 202201305A3). To increase the clinical applicability of KDpredictor in cases where certain blood features may be unavailable, we later performed SHAP analysis on the constructed full KDpredictor model to identify its top five predictive features. Then, a reduced model based solely on these five features was subsequently developed to accommodate data with partial blood test information.

### 2.2 The collected lab features

We gathered lab features from routine blood test results in this study to support KD risk identification. The information collected from medical records included demographic characteristics (age and gender), complete blood count with differential count (CBC/DC), C-reactive protein (CRP), and alanine aminotransferase (ALT) (See Table 1 for a detailed feature list). Among the demographic variables, only age was incorporated into the final KD identification model. For KD patients, laboratory blood test results were obtained within 24 hours prior to the administration of the first intravenous immunoglobulin (IVIG) treatment. Recognizing that the normal reference ranges for hemoglobin, eosinophil, and platelet count vary by age group [23], [24], [25], [26], we computed age-calibrated scores for these three features using population statistics (mean and standard deviation) derived from a reference dataset of approximately 100,000 children from Chang Gung Memorial Hospital. A positive age-calibrated score indicates a value above the age-group average. All laboratory blood tests included in this study are part of standard pediatric emergency care and can typically be processed and interpreted by clinicians within one hour after blood sample collection. The summary statistics, including averages and standard errors for KD and FC groups, are presented in Table 1.

**Table 1:** The lab features used in KDpredictor. The values are represented in the form of mean ± standard error.

Feature Name	Unit	Calculation	Febrile Controls	Kawasaki disease patients
Age	month	-	24.17 ± 0.10	20.93 ± 0.40
Gender	-	(not used in the final model)	M: 25,439, F: 19,835	M: 1,077, F: 708

RBC (Red Blood Cell)	million/μL	-	4.53 ± 0.002	4.26 ± 0.01
Hemoglobin	g/dL	-	11.87 ± 0.006	10.92 ± 0.03
Hematocrit	%	-	35.41 ± 0.02	32.82 ± 0.08
MCV (Mean Corpuscular Volume)	fL	-	78.65 ± 0.03	77.47 ± 0.15
MCH (Mean Corpuscular Hemoglobin)	pg/cell	-	26.39 ± 0.01	25.80 ± 0.06
MCHC (Mean Corpuscular Hemoglobin Concentration)	gHb/dL	-	33.52 ± 0.005	33.27 ± 0.02
Platelets	1000/μL	-	277.33 ± 0.50	356.37 ± 3.29
ALT/GPT (Alanine AminoTransferase)	U/L	-	26.26 ± 0.34	80.28 ± 3.70
CRP (C-reactive protein)	mg/L	-	23.08 ± 0.17	90.89 ± 1.68
WBC (White Blood Cell)	1000/μL	-	10.70 ± 0.03	14.15 ± 0.12
Band	%	-	0.52 ± 0.008	1.55 ± 0.08
Segment	%	-	53.07 ± 0.09	58.75 ± 0.36
Lymphocyte	%	-	35.64 ± 0.08	29.65 ± 0.33
Monocyte	%	-	8.83 ± 0.02	6.16 ± 0.08
Eosinophil	%	-	0.98 ± 0.007	3.09 ± 0.07
Basophil	%	-	0.24 ± 0.002	0.21 ± 0.009
Band count	/μL	WBC x Band (%)	60.95 ± 1.03	223.94 ± 12.10
Segment count	/μL	-	5989.55 ± 19.82	8510.20 ± 100.11
Lymphocyte count	/μL	-	3518.45 ± 10.09	4019.12 ± 51.08
Eosinophil count	/μL	-	102.98 ± 0.89	421.82 ± 10.47
Basophil count	/μL	-	23.48 ± 0.18	27.62 ± 1.29
Age-calibrated Platelet score	-	age-corrected score	0.12 ± 0.005	1.06 ± 0.04
Age-calibrated Eosinophil score	-	age-corrected score	-0.09 ± 0.004	1.37 ± 0.05
Age-calibrated Hemoglobin score	-	age-corrected score	-0.30 ± 0.005	-1.28 ± 0.03

### 2.3 The workflow of KDpredictor

**Fig. 1:**
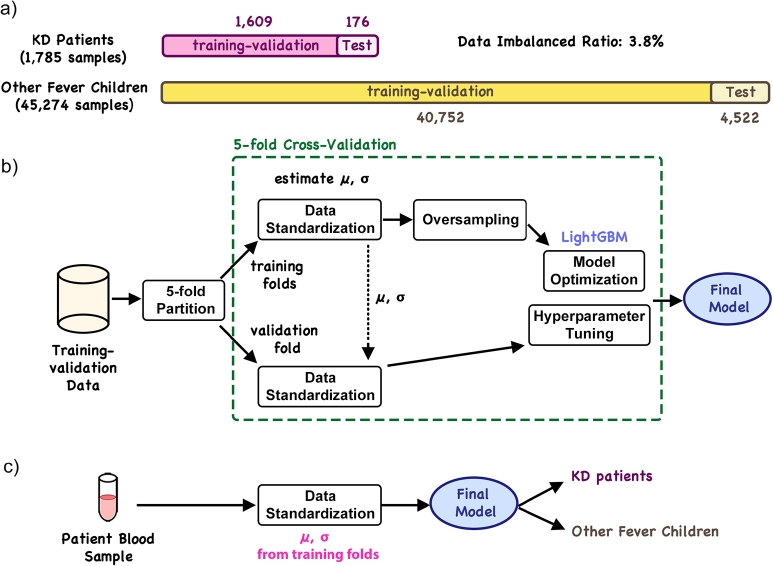
The construction of KDpredictor. (a) Data partition. (b) The construction steps of KDpredictor. (c) KD identification by KDpredictor.

The training and inference workflows of KDpredictor are illustrated in Fig. 1. To systematically develop the tool using machine learning algorithms, we first divided the ground-truth data of KD and other fever objects into a training-validation set and a test set (Fig. 1-a). One-tenth of the data was reserved as an untouched test set, which was excluded from all stages of training. This test set was used exclusively for evaluating the generalization performance of the final model and for fair comparison with other existing KD identification tools. The training-validation set consisting of the remaining data was used for learning algorithm selection, model tuning, and optimization [27]. In this research, we employed the five-fold cross-validation strategy to systematically construct the model on the training-validation set. In brief, the training-validation set is partitioned into five different folds. For each iteration, one fold was treated as the validation set for current performance estimation, and the other four folds were used for model parameter optimization. After five iterations, where each time one fold is treated as the validation set, the best-suited hyperparameters were selected based on the average of a predefined performance metric. We adopted F1 (see “Evaluation metrics” Section) as the indicator metric.

To eliminate the data range effect that can adversely bias the overall model performance [28], we standardized all given blood features prior to model training:

$$\mathrm{nor}\_\mathrm{valu}e_{\mathrm{ij}}=\frac{x_{ij}-\mu_{j}}{\sigma_{j}},$$

where $x_{ij}$ is the *j*th feature of the *i*th patient sample, $\mu_{j}$ is the estimated population mean of the *j*th feature, and $\sigma_{j}$ is the estimated population standard deviation of the *j*th feature. Since $\mu_{j}$'s and $\sigma_{j}$'s are also hyperparameters of the overall method, these values were estimated on the training folds within each cross-validation iteration. And the final $\mu_{j}$'s and $\sigma_{j}$'s were selected from the best cross-validation iteration and fixed for the prediction process.

After standardizing the feature values, we trained a machine-learning model to distinguish KD patients from FC cases based on these inputs. As described in the “The study population” section, the number of FC samples significantly exceeded that of KD patients, resulting in a substantial class imbalance. To deal with this issue, we applied independent oversampling (sampling with replacement) to the minority class (KD patients) within each training and validation fold during every iteration of the cross-validation process (Fig. 1-b). To ensure proper model fitting and avoid overfitting, we adopted the learning curve technique in the cross-validation process. For every cross-validation round, three types of learning curves were generated to help evaluate the data imbalance issue mitigated by oversampling: the performance curves from the oversampled training folds, the oversampled validation fold, and the original validation fold. The average learning curve of the oversampled training folds and the corresponding curve of the oversampled validation folds were used to assess model fitting, while the average learning curve formed by the original validation folds provided an unbiased estimate of model performance. Following a comparative evaluation of candidate machine learning algorithms, the final algorithm selected in building the KD classification model was LightGBM, a gradient-boosted decision tree method. The final model tuning hyperparameters were: learning rate = 0.2, minimum data in a leaf = 10, maximum depth = 5, and number of leaves = 5.

The prediction process of KDpredictor is illustrated in Fig. 1-c. During inference, the input blood features are first standardized using the $\mu_{j}$ and $\sigma_{j}$ of each feature, where $\mu_{j}$ and $\sigma_{j}$ were estimated exclusively from the training-validation set to avoid data snooping. These features are then passed into the obtained model based on LightGBM to obtain the probability that the given blood sample is from a KD patient. In the final version of KDpredictor, a fixed probability threshold of 0.5 is suggested for classifying a sample as KD-positive.

### 2.4 Evaluation metrics

In this study, we evaluated the performance of various KD identification tools using the confusion matrix framework [29]. In the confusion matrix of KD identification, we define TP (true positive) as the number of correctly identified KD patients, FP (false positive) as the number of febrile controls misclassified to be KD patients, FN (false negative) as the number of missed KD patients, and TN (true negative) as the number of correctly recognized febrile children that do not have KD. Based on these definitions, the following evaluation metrics are calculated:

$$\mathrm{Recall}=\frac{TP}{TP+FN},\;\mathrm{Specificity}=\frac{TN}{FP+TN}=1-\mathrm{FPR},$$

$$\mathrm{Precision}=\frac{TP}{TP+FP},\;\mathrm{F1}=\frac{2⁎\mathrm{Precision}⁎\mathrm{Recall}}{\mathrm{Precision}+\mathrm{Recall}},$$

$$LR^{+}=\frac{\mathrm{Recall}}{1-\mathrm{Specificity}},\;LR^{-}=\frac{1-\mathrm{Recall}}{\mathrm{Specificity}}.$$

Since the F1 score reflects the trade-offs between precision and recall [30], it was selected as the primary performance indicator in the learning curve analysis. All performance metrics, including precision, recall, specificity, F1 score, and LR (likelihood ratio) values, were computed using a fixed prediction threshold of 0.5, which was considered a fair and clinically meaningful cutoff. In the above equations, likelihood ratios are the overall measurements of the sensitivity values and the specificity values. Specifically, positive likelihood ratios depict the likelihood of a positive test on a patient with KD compared with the likelihood of a positive test on other fever conditions, helping understand KD risks by neglecting the effect of prevalence.

To further evaluate the intrinsic capabilities of each KD identification method, we resorted to receiver operating characteristic (ROC) curves [28]. Given the significant class imbalance between KD and febrile control (FC) groups in the dataset, we also considered precision-recall (PR) curves [31] for a more informative analysis regarding the prevalence of KD. The ROC curve plots recall (sensitivity) against false positive rate (FPR) across various adjusted thresholds. An ROC curve closer to the upper-left corner indicates superior model performance, demonstrating that the tool can still have high recall when the FPR is controlled tightly. The area under the ROC curve (auROC) quantifies this overall performance. Similarly, the PR curve illustrates the trade-off between precision and recall across different thresholds. The area under the precision-recall curve (auPRC) provides an aggregated measure of model effectiveness in identifying KD under imbalanced conditions. In both cases, higher auROC and auPRC values indicate better model performance.

## 3 Results

### 3.1 The performance of KDpredictor

**Fig. 2:**
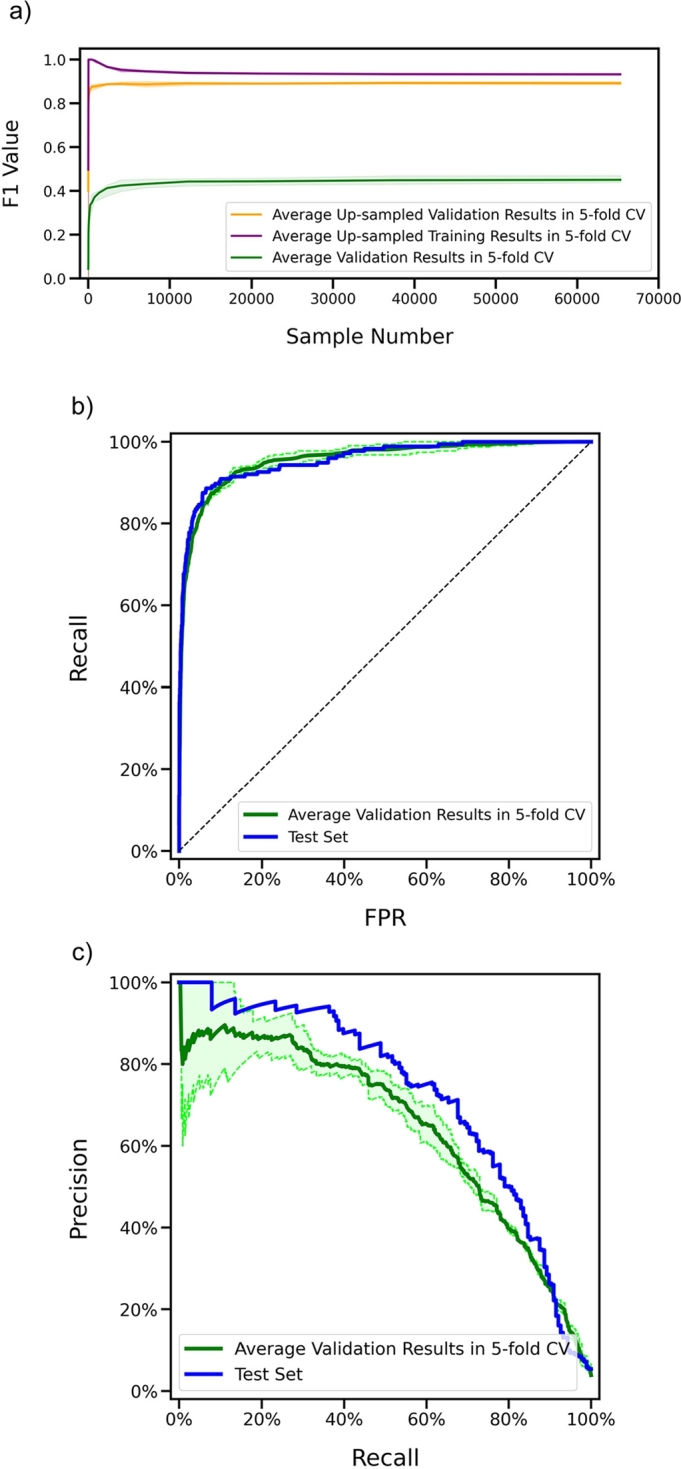
The learning curves and performance curves of KDpredictor. The shaded area represents the range of the performance of the 5 iterations in 5-fold CV. (a) The learning curves. (b) The ROC curves of the validation results and the test result. (c) The PR curves of the validation results and the test results.

We chose the suitable hyperparameters and optimized the parameters of KDpredictor on the prepared training-validation set using five-fold cross-validation. To enforce model convergence, we employed a sample number-based learning curve approach [32]. As illustrated in Fig. 2, the average learning curves for both the over-sampled training and validation folds converged to similar F1 values as the number of samples increased, indicating that the model was well-fitted. On the 5-fold validation sets, KDpredictor obtained an average auROC of 95.6% and an average auPRC of 64.5% (Fig. 2-b and 2-c). The corresponding average validation recall and F1 were 0.87 and 0.45, respectively. These performance values are substantially better than random guessing in the context of the low prevalence of KD. Other performance metrics are listed in Table 2. From these validation results, KDpredictor was verified to score high KD identification performance.

**Table 2:** The performance summary of KDpredictor on the five-fold validation sets and the reserved test set.

Dataset	auROC	auPRC	F1	Precision	Recall	Specificity	LR+	LR-
5-fold Val	95.6%	64.5%	0.45	0.31	0.87	0.92	11.12	0.14
Test	95.7%	72.4%	0.45	0.30	0.89	0.92	11.17	0.12

To assess the generalization of KDpredictor on unseen KD patient samples, we evaluated KDpredictor on the reserved test set. The test set results yielded an auROC of 95.7% and an auPRC of 72.4% (See Fig. 2-b and 2-c), closely aligning with the average validation performance and demonstrating the model's excellent generalization. The test F1 value and recall of KDpredictor also followed similar trends (See Table 2). Overall, these findings confirm that KDpredictor achieves high accuracy in KD risk estimation and generalizes effectively to new blood test data from febrile patients.

As indicated in the “Workflow of KDpredictor” section, we recommend using a prediction threshold of 0.5 in KDpredictor. This default threshold yields a test recall of 0.89 and a test specificity of 0.92. In ROC curves, an ideal classifier has 100% recall and specificity, which corresponds to the top-left corner of the ROC curve plots. As shown in Fig. 2-b, the recall and (1-specificity) values obtained under the threshold of 0.5 is around the closest point to the ideal classifier. Hence, the default threshold of 0.5 recommended for KDpredictor not only maintains a strong balance between recall and specificity but also represents the near-optimal cutoff as suggested by the ROC curve analysis.

### 3.2 Evaluation of using different learning algorithms in KDpredictor

In designing KDpredictor, we ultimately selected LightGBM as the learning algorithm for identifying KD-related features, as indicated in the “Model” component of Fig. 1. To justify this choice, we systematically investigated several widely used feature-based machine learning algorithms through five-fold cross-validation. The algorithms assessed included Linear Discriminant Analysis (LDA), AdaBoost, LightGBM, Extreme Gradient Boosting (XGBoost), Random Forest, Support Vector Machine (SVM), Logistic Regression, and Multi-layer Perceptron (MLP). All learning algorithms were implemented using the Scikit-Learn Python package [33]. Since selecting the most appropriate learning algorithm constitutes a major step in model hyperparameter tuning [32], each algorithm was integrated into the KDpredictor pipeline and evaluated on the five-fold validation partitions. To ensure proper convergence and prevent under- or overfitting, the training process for each integrated algorithm for the KDpredictor pipeline was also monitored using the sample-based learning curve technique.

In Table 3, we summarize the validation results of these model variants consisting of different learning algorithms for the KDpredictor pipeline. While the auROC values were relatively consistent across model variants, substantial differences were observed in the auPRC values. Given the low prevalence of KD among febrile patients, auPRC is a more appropriate metric for evaluating a model's effectiveness in handling class imbalance. In general, model variants based on boosting or ensemble techniques (such as LightGBM, AdaBoost, XGBoost, and Random Forest) as well as deep neural networks can outperform simpler models like LDA and logistic regression by at most 15% in auPRC. Among the high-performing models, LightGBM was selected as the core learning algorithm for KDpredictor due to its competitive performance, efficient training speed, and reduced memory consumption. Based on this comparative analysis, we conclude that LightGBM is an optimal choice for KDpredictor's algorithmic foundation.

**Table 3:** The performance evaluation of different learning algorithms inserted into the KDpredictor pipeline on the five-fold validation folds.

Learning Algorithm	auROC	auPRC	F1	Precision	Recall	Specificity	LR+	LR-
LightGBM (KDpredictor)	95.60%	64.50%	0.45	0.31	0.87	0.92	11.12	0.14
AdaBoost	95.20%	63.00%	0.44	0.3	0.86	0.92	10.69	0.15
XGBoost	95.60%	63.50%	0.42	0.28	0.88	0.91	9.72	0.13
MLP	95.60%	65.50%	0.45	0.3	0.86	0.92	10.86	0.15
Random Forest	94.60%	58.50%	0.4	0.26	0.87	0.9	8.84	0.15
SVM	94.80%	60.70%	0.45	0.3	0.85	0.92	11.05	0.17
Logistic Regression	94.20%	49.20%	0.39	0.25	0.85	0.9	8.55	0.16
LDA	93.60%	48.90%	0.4	0.26	0.82	0.91	8.95	0.2

### 3.3 Comparison with related works

Two previous studies have incorporated blood test features with clinical signs for KD identification: the works by Lam et al. [18] and Ling et al. [19]. Lam et al. aimed to differentiate KD from multisystem inflammatory syndrome in children (MIS-C) using a combination of clinical signs and laboratory blood features. Their selected blood parameters included white blood cell count, neutrophils (%), bands (%), lymphocytes (%), atypical lymphocytes (%), monocytes (%), eosinophils (%), neutrophil count, band count, lymphocyte count, hemoglobin concentration, platelet count, erythrocyte sedimentation rate, CRP, ALT, albumin, sodium, and *γ*-glutamyl transferase. Ling et al. focused on distinguishing KD from other febrile illnesses using both clinical parameters and laboratory blood features. The adopted blood test features by Ling et al. include eosinophils (%), CRP, erythrocyte sedimentation rate, white blood cell count, monocytes (%), *γ*-glutamyl transferase, platelet (%), ALT, neutrophils (%), immature neutrophils (%), lymphocytes (%), and hemoglobin concentration Z-score. In this study, which emphasizes the use of only objective laboratory blood samples, we adopted only the blood features selected by Lam et al. (originally used with linear discriminant analysis) and Ling et al. (used with neural networks) for comparison. We retrained their models based on their selected and available blood features and compared the performance of the retrained models with KDpredictor. Convergence of the retrained models was confirmed using the learning curve technique. It is important to note that although various specialized biomarkers for KD diagnosis have been proposed, they are not routinely measured in clinical evaluations of febrile children. This study specifically focuses on leveraging widely available routine blood tests, namely complete blood count with differential count (CBC/DC), CRP, and ALT/GPT, for KD risk estimation. These blood tests are typically performed in febrile pediatric patients, enhancing the clinical applicability of the proposed KDpredictor tool. Biomarkers not typically included in standard evaluations were beyond the scope of this research and thus not included in the comparison.

**Fig. 3:**
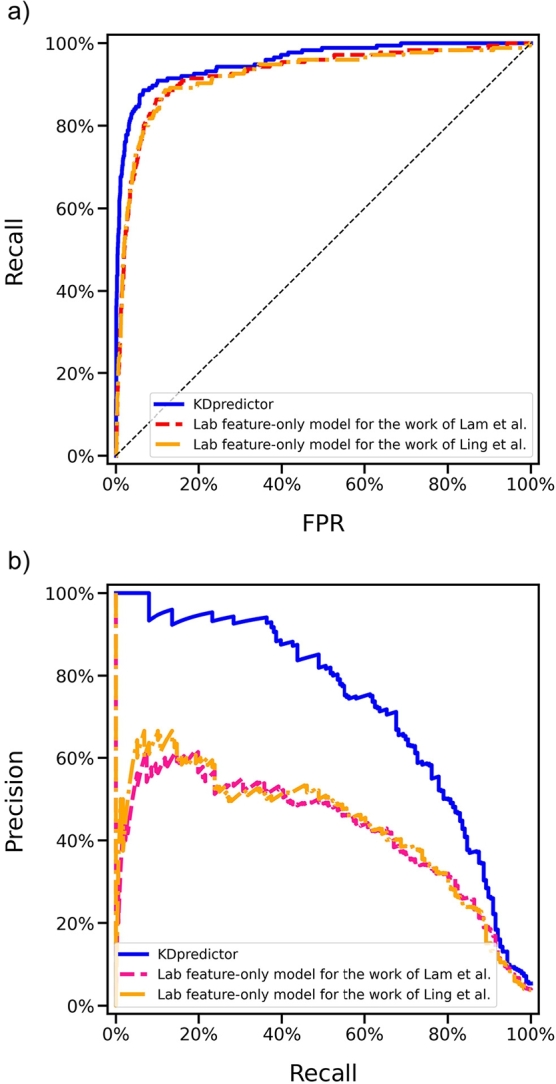
The (a) ROC and (b) PR curve comparison among KDpredictor and related works on the test set.

The comparison among KDpredictor and the retrained models using features selected in their original works was performed on the reserved test set. We summarized the performance comparison in Fig. 3 and Table 4. Compared with the retrained model based on lab features used by Ling et al., over 3.4% (95.7% vs. 92.3%), 29.3% (72.4% vs. 43.1%), and 5% (89% vs. 84%) improvements in auROC, auPRC, and recall values, respectively, were observed by KDpredictor. Similarly, when compared with the retrained model derived from the lab features reported by Lam et al. (auROC = 92.7%, auPRC = 42.1%, recall = 89%), KDpredictor showed performance gains of 3% in auROC and 30.3% in auPRC. In summary, KDpredictor consistently outperforms models based on previously published blood feature sets, achieving at least a 3% improvement in auROC and a 29.3% improvement in auPRC. These findings support the conclusion that KDpredictor leverages the most informative and relevant routine blood features for effective KD identification, thus providing superior performance relative to prior approaches.

**Table 4:** The performance comparison among different lab feature sets used in related works on the prepared test set.

Related Works	auROC	auPRC	F1	Precision	Recall	Specificity	LR+	LR-
KDpredictor	95.7%	72.4%	0.45	0.30	0.89	0.92	11.17	0.12
Lab feature-only model by Ling et al.	92.3%	43.1%	0.37	0.24	0.84	0.90	8.02	0.18
Lab feature-only model by Lam et al.	92.7%	42.1%	0.33	0.21	0.89	0.87	6.64	0.13

### 3.4 Independent tests for KD patients in different clinical cohorts collected during the COVID-19 period

To independently evaluate the performance of KDpredictor, we conducted a retrospective confirmation test using KD patient data collected during the COVID-19 pandemic, aiming to assess its robustness across varying clinical contexts. Three distinct KD cohorts from East Asia were included: (1) 142 IVIG-treated patients from Chung Gung Medical Foundation, Taiwan (collected between January 2020 and November 2023; median age approximately 23 months; gender data unavailable); (2) 141 IVIG-treated patients from Chi Mei Medical Center, Taiwan (collected between May 2008 and August 2023; median age approximately 20 months; 83 males and 58 females); and (3) 12 patients from The First People's Hospital of Jingzhou, China (collected between January 2015 and January 2024; median age approximately 23 months; 9 males and 3 females). Complete blood test features were available for all patients, enabling the application of the full KDpredictor model without modification in this retrospective confirmation test. As these cohorts consisted solely of confirmed KD cases, only recall could be evaluated. KDpredictor achieved recall rates of 90.9%, 83.7%, and 91.7% for the first, second, and third independent cohorts, respectively. These findings confirm that KDpredictor effectively identifies KD cases across independent clinical settings using only blood test features, supporting its potential utility as a reliable diagnostic aid even in the early stages of the disease when clinical symptoms may not yet be apparent.

### 3.5 The top distinguishing features of KDpredictor

**Fig. 4:**
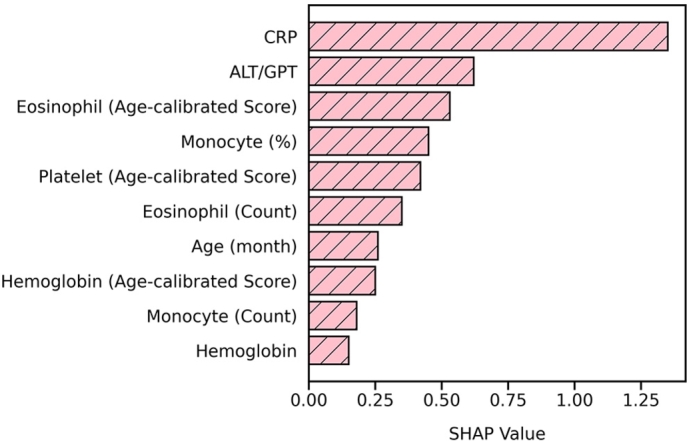
The top 10 identifying lab features of KDpredictor from SHAP analysis.

Traditionally, most machine learning-based diagnostic tools have operated as black boxes, offering only a predicted probability that, when compared with a predefined threshold, determines whether a sample is classified as diseased or normal. However, such opaque decision-making processes often raise concerns in clinical applications due to the lack of interpretability and transparency [34]. To address this issue, researchers have developed explainable artificial intelligence (XAI) methods that aim to elucidate the reasoning behind machine learning or deep learning model predictions [35]. One widely adopted XAI method is SHAP (SHapley Additive exPlanations) [36], which computes SHapley values (or simply SHAP values) to quantify the contribution of each input feature to the final prediction. A higher SHAP value indicates a stronger influence of the corresponding feature on the model's prediction. Using SHAP analysis on the collected training-validation set in this research, we identified the top five distinguishing features used by KDpredictor (See Fig. 4): C-reactive protein (CRP), alanine aminotransferase (ALT), age-calibrated eosinophil score, monocytes (%), and age-calibrated platelet score. A similar feature importance pattern was also observed on the test set, confirming their consistency. These five features are the most critical factors that KDpredictor relies on when distinguishing KD patients from febrile controls.

**Fig. 5:**
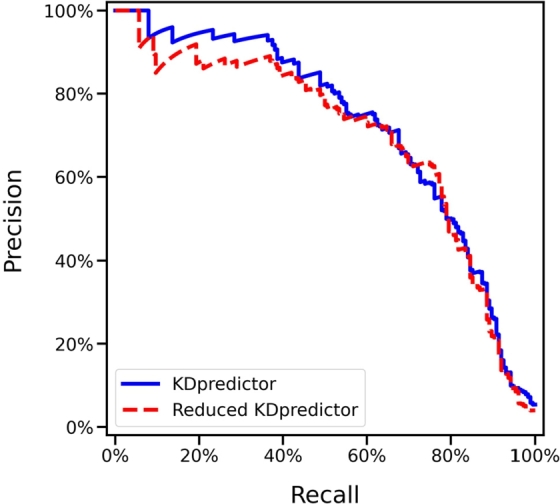
The PR curve comparison between KDpredictor and the reduced KDpredictor model on the test set.

We further constructed a model using only these five blood test features (CRP, ALT, age-calibrated eosinophil score, monocyte percentage, and age-calibrated platelet score) to see if we can provide a further reduced version of KDpredictor (called the reduced KDpredictor model in our context). The reduced KDpredictor model was retrained on the training-validation set using five-fold cross-validation, with convergence and proper model fitting verified through the learning curve analysis. As shown in Fig. 5, the reduced model achieved an auPRC of 69.5% on the test set, representing only a modest 2.9% decrease compared with the full-featured KDpredictor (auPRC = 72.4%). A similar trend was observed in the auROC values, as summarized in Table 5. These results suggest that KD identification can be effectively performed using a compact set of key blood features, with only minimal performance auPRC trade-off. The reduced model thus holds promise for rapid and efficient KD screening, particularly in emergency or resource-limited clinical settings.

**Table 5:** The test set performance comparison between the full-feature KDpredictor and the reduced KDpredictor model using only the top 5 identifying features.

Model	auROC	auPRC	F1	Precision	Recall	Specificity	LR+	LR-
KDpredictor	95.7%	72.4%	0.45	0.30	0.89	0.92	11.17	0.12
Reduced KDpredictor	94.2%	69.5%	0.44	0.29	0.89	0.92	10.39	0.124

### 3.6 Exploring the prediction of KD through the use of various blood test prescriptions

In clinical practice, different blood test items are prescribed under varying panels. Some common blood test prescriptions are categorized as follows: (1) CBC-only prescription, which provides 10 items including WBC, RBC, hemoglobin, Hematocrit, MCV, MCH, MCHC, RDW-SD, platelets, and RDW-CV. (2) Prescription for CBC and WBC/DC (WBC differential count), which includes CBC items along with lymphocyte (%), segment (%), eosinophil (%), basophil (%) and band form (%). (3) Prescription for CBC, WBC/DC, and ALT/GPT, resulting in 16 items. (4) Prescription for CBC, WBC/DC, and CRP, leading to 16 items. (5) Full prescription with CBC, WBC/DC, and biochemistry tests, which combines CBC, WBC/DC, and biochemistry test results, yielding 17 items (the default model reported for KDpredictor). As demonstrated in earlier sections, KDpredictor achieves strong performance using the full prescription items. To evaluate its adaptability to more limited blood test prescription scenarios, we trained additional KDpredictor variants based on each of the above-categorized prescription types using the same LightGBM learning algorithm. Patient age (in months) was included as an additional feature in all models. The convergence of model variants using items available in different prescriptions was verified using sample-number-based learning curves, and their performance metrics were then evaluated on the reserved test set.

As shown in Table 6, the full-feature KDpredictor (model based on the prescription of CBC, WBC/DC, and full biochemistry tests) achieved an auROC of 95.7%, auPRC of 72.4%, and recall of 0.89. In contrast, the model using only CBC features showed moderate performance, with auROC/auPRC/recall of 85.5%/27.7%/0.78. Notably, adding WBC/DC features substantially improved performance; models with at least CBC and WBC/DC achieved auROC, auPRC, and recall values of 91.6%, 51.1%, and 0.84, respectively. The addition of partial or full biochemistry data (e.g., CRP or ALT) further enhanced KD identification performance. Other test set performances (See Table 6) of these model variants reveal the same trend. This consistent trend across model variants highlights the trade-off between diagnostic accuracy and the extent (and cost) of laboratory blood tests. In summary, these KDpredictor variants demonstrate that effective KD risk estimation is possible under a range of commonly prescribed test panels. This flexibility allows clinicians to adapt KDpredictor in settings where only partial test results are available, thereby supporting more accessible and scalable KD screening in diverse clinical environments.

**Table 6:** The test performance summary of models based on features from different blood test prescriptions.

Model	auROC	auPRC	F1	Precision	Recall	Specificity	LR+	LR-
Prescription for CBC	85.5%	27.7%	0.21	0.12	0.78	0.79	3.6	0.28
Prescription for CBC and WBC/DC	91.6%	51.1%	0.31	0.19	0.84	0.86	6.0	0.19
Prescription for CBC, WBC/DC, and ALT	93.4%	64.1%	0.37	0.23	0.85	0.89	7.9	0.17
Prescription for CBC, WBC/DC, and CRP	94.9%	63.2%	0.41	0.27	0.89	0.91	9.3	0.13
Prescription for CBC, WBC/DC, CRP, and ALT	95.7%	72.4%	0.45	0.30	0.89	0.92	11.2	0.12

### 3.7 KDpredictor is not age- or gender-biased for children under seven years old

It is well-established that several blood test parameters, such as the complete blood count (CBC) and alanine aminotransferase (ALT), can vary with age. To mitigate age-related confounding effects, our study design restricted the cohort to patients under seven years old, a subgroup in which KD is most commonly observed. We also included “age” as an input feature in KDpredictor to allow the model to account for potential age-related variability in KD risk estimation. As shown in the SHAP analysis presented in the section “The Top Distinguishing Features of KDpredictor,” the “age” feature does not exhibit dominant model importance relative to other features. This suggests that KDpredictor primarily relies on more biologically specific blood features, rather than age alone, when estimating KD risk.

To further investigate the potential bias of age and gender inherent in the collected dataset and thus the constructed model, we adjusted the training-validation set to be age-controlled and gender-matched. Specifically, we re-sampled the negative cases (i.e., febrile patients without KD) to match its age and sex distribution to those of the KD cases, thereby controlling the demographic imbalance in the training-validation set. Using this matched training-validation set, we trained a new KD prediction model, referred to as the background-matched model, following the same methodology described in the “The Performance of KDpredictor” section. Convergence of the background-matched model was again verified using the learning curve technique. We then compared the performance of the background-matched model with the original KDpredictor model on the reserved test set. As summarized in Table 7, both models exhibited comparable auROC and auPRC values, suggesting that KDpredictor is not biased by age or gender within the studied population. Accordingly, we conclude that KDpredictor provides reliable KD risk estimation for children under seven years of age, regardless of demographic variation.

**Table 7:** The performance comparison between KDpredictor and the background (gender and age) matched model on the prepared test set.

Related Works	auROC	auPRC	F1	Precision	Recall	Specificity	LR+	LR-
KDpredictor	95.7%	72.4%	0.45	0.30	0.89	0.92	11.17	0.12
Background-matched model	95.5%	72.7%	0.46	0.31	0.89	0.92	11.31	0.12

### 3.8 Both the over-sampling and under-sampling techniques can lessen the data imbalance issue in KD risk estimation

In this study, we addressed the class imbalance inherent in the dataset by applying simple random over-sampling. This technique increases the representation of positive samples, i.e., KD patient cases, during training, thereby guiding the model to better recognize underrepresented KD instances. The degree of emphasis on positive samples can be controlled by adjusting the sampling ratio with replacement. Alternatively, under-sampling reduces the number of negative samples (febrile controls) to achieve a similar effect by balancing the class distribution through suppression rather than amplification. These two methods for handling the problem of data imbalance may suit different tasks [37]. To assess the applicability of under-sampling for training KDpredictor, we also reported a model variant using under-sampled negative cases within the training-validation set. The convergence of this under-sampled model was also verified using the learning curve analysis. Performance metrics for both the over-sampled (i.e., KDpredictor) and under-sampled models on the reserved test set are summarized in Table 8. As shown, the under-sampled model achieved performance comparable to the original over-sampled model (KDpredictor). These results suggest that KDpredictor is robust to the choice of class balancing strategy, and that both over-sampling and under-sampling are viable approaches for managing data imbalance in this context.

**Table 8:** The performance comparison between models trained by different class balancing strategy on the prepared test set.

Related Works	auROC	auPRC	F1	Precision	Recall	Specificity	LR+	LR-
KDpredictor	95.7%	72.4%	0.45	0.30	0.89	0.92	11.17	0.12
Under-sampled model	95.6%	69.6%	0.40	0.26	0.90	0.90	9.0	0.11

## 4 Discussions

The clinical diagnosis of KD traditionally relies on five major diagnostic criteria, along with additional signs such as Bacillus Calmette-Guérin vaccine site induration, arthritis, diarrhea, vomiting, and headache. Laboratory evaluations for KD often include CBC/DC, CRP, ESR, ALT/GPT, sodium, *γ*GT, albumin, and urine routine. In this study, we developed KDpredictor using only objective laboratory data (CBC/DC, CRP, and ALT) to enable accurate KD risk estimation without relying on subjective clinical symptoms or additional biomarkers. The required laboratory tests can be performed using just two blood collection tubes, requiring a total blood volume of approximately 1.5 ml or less, making it feasible for rapid application in pediatric emergency settings. Notably, based on our literature review, this study represents the inaugural application of age-calibrated scores for platelet, hemoglobin, and eosinophil to construct an algorithm for identifying KD from other fever illnesses. This approach demonstrates high performance in terms of auROC and auPRC, offering a clinically practical and data-driven solution for early KD detection, as further discussed in the following.

### 4.1 Clinical analysis of the top distinguishing features in KDpredictor

Among the top ten features identified by KDpredictor (See Fig. 4), eosinophil (both unit count and age-calibrated scores) and hemoglobin levels are particularly noteworthy. We additionally highlight the clinical significance of these indicators, demonstrating that KDpredictor not only provides accurate predictions but also offers valuable biological insights into KD.

It is believed that eosinophils are central to type 2 immune responses and are commonly involved in hypersensitivity reactions, allergic diseases, and parasitic infections [38]. In 2002, Terai et al. first reported peripheral blood eosinophilia and eosinophil accumulation in coronary microvessels of acute KD patients [39]. Our group later corroborated these findings, confirming eosinophilia in the acute phase of KD and increased eosinophil levels after IVIG administration [40]. Importantly, post-IVIG eosinophil levels were found to inversely correlate with IVIG resistance [40] and the development of coronary artery lesions (CALs) [25].

Liu et al. further emphasized the diagnostic relevance of eosinophilia in a nomogram-based model for distinguishing KD from other febrile illnesses, where eosinophils were among the most discriminative features [41]. In addition, patients with KD have been shown to have a higher incidence of allergic diseases such as allergic rhinitis and asthma [42]. In these allergic diseases, two distinct eosinophil subtypes have been categorized: lung-resident eosinophils that maintain homeostasis and inflammatory eosinophils that reflect type 2 tissue inflammation [43]. Chang et al. later pointed out an elevated expression of SELL, a marker of lung-resident eosinophils, in children with KD, suggesting a role for eosinophil subtypes in KD pathogenesis [44]. While these findings point to a potential immunological mechanism, further research is needed to fully elucidate the functional role of eosinophils in KD pathogenesis. All these recent studies on eosinophils in KD suggest that eosinophils in blood samples are reasonably indicative of KD.

Hemoglobin is another critical marker highlighted by KDpredictor. Decreased hemoglobin levels are commonly observed in KD and are associated with acute systemic inflammation and hemolysis [45], [26]. Hemoglobin has also been identified as a useful predictor in distinguishing KD from conditions such as sepsis [46], KD shock syndrome, and toxic shock syndrome [47]. Taken all together, these findings affirm that the key features identified by KDpredictor, particularly eosinophil and hemoglobin levels, are not only diagnostically informative but also clinically meaningful. The inclusion enhances the interpretability of the model and supports its utility in both diagnosis and the ongoing study of KD pathophysiology.

### 4.2 Potential integration of KDpredictor into the clinical practice

KDpredictor is a blood test-based tool developed to estimate the risk of KD in febrile children. It is intended to assist, rather than replace, clinical decision-making. Clinical judgment remains paramount. To support the integration of KDpredictor into clinical workflows, the following usage strategy may be applicable. When the estimated KD risk of a febrile child exceeds 0.5 (i.e., risk probability ≥ 0.5), physicians should initiate daily monitoring for the emergence of major and minor KD symptoms and signs until his/her fever subsides or the clinical possibility of KD can be confidently excluded for the child. In this context, KDpredictor functions as a clinical alert system to help draw attention to cases that may warrant closer observation for KD. To further evaluate the practical utility of the tool, we have designed a prospective study in which physicians may request a KD risk assessment when complete blood count with differential count (CBC/DC), C-reactive protein (CRP), and liver enzyme levels (ALT/GPT) are available in the emergency department or outpatient clinic. The outcomes of this ongoing prospective study will be reported in future work.

### 4.3 Issues of false positives and false negatives by KDpredictor

KDpredictor was developed to help estimate the risk of KD using only routine blood test results. Through carefully designed feature processing and model optimization, KDpredictor achieves a notable improvement of at least 39.3% in auPRC (auPRC = 72.4%) compared with existing methods (see Fig. 3), showing a substantial advancement in identifying KD in low-prevalence settings. The auPRC, which reflects the model's ability to distinguish positive cases under class imbalance, is a particularly relevant metric for rare diseases such as KD. This significant auPRC gain highlights KDpredictor's enhanced capability in risk evaluation compared with prior tools. However, from a theoretical perspective grounded in learning theory, the trade-off between precision and recall is constrained by the prevalence of the target condition. Specifically, when maintaining a high recall rate, the achievable precision is limited by the low prevalence of KD in the clinical population. Thus, despite KDpredictor's 39.3% improvement in auPRC, its precision remains inherently constrained, leading to an unavoidable number of false positives in practice. Future prospective studies are warranted to further investigate the sources of false positives and false negatives generated by KDpredictor, which will help guide model refinement and inform clinical integration strategies for more accurate and practical KD risk assessment.

Although several biomarkers, such as N-terminal pro-brain natriuretic peptides (NT-proBNPs) [48], have been identified as potentially useful for KD diagnosis, they are not routinely included in the standard laboratory evaluation of febrile children. Moreover, even when some markers, such as NT-proBNP levels, are incorporated into diagnostic models, the challenges of false-positive and false-negative predictions remain unresolved. As such, KDpredictor, like any diagnostic aid, is not immune to these limitations. To mitigate diagnostic uncertainty, repeated blood test monitoring and clinical assessment of KD-specific symptoms remain essential. These complementary approaches can help reduce misclassification and improve the overall diagnostic precision in real-world clinical practice.

Currently, KDpredictor is designed as an initial risk assessment tool for children presenting with fever or suspected KD, with the goal of aiding early detection and reducing the risk of coronary artery lesions. It is not intended to serve as a definitive diagnostic tool. The final diagnosis of KD and the decisions to prescribe IVIG should still be made by a qualified physician based on established diagnostic criteria. KDpredictor may be used to raise clinical suspicion or serve as supportive evidence in the diagnostic process. When the estimated KD risk exceeds a threshold of 0.5 (i.e., risk probability ≥ 0.5), it is recommended that physicians closely monitor the patient for evolving major and minor KD signs and symptoms on a daily basis until the fever resolves or KD can be confidently excluded for the patient. In summary, KDpredictor is intended to function as an alert system to prompt additional clinical attention in febrile children potentially at risk for KD. While it provides valuable decision support, it is not a substitute for physician judgment. Its integration into clinical workflows may help overcome the inherent challenges of diagnosing low-prevalent KD, particularly by improving early recognition and prioritizing follow-up evaluation.

### 4.4 Limitation of KDpredictor

Finally, this study aims to identify the risk of KD for a febrile child using only routine blood tests without detailed clinical symptoms. The collected febrile control (FC) group may include a range of other pediatric symptoms such as juvenile idiopathic arthritis (JIA), multi-system inflammatory syndrome in children (MIS-C), adenovirus infections, and streptococcus infections. However, specific diagnostic sub-classifications within the FC group were not available. Patients were only followed to confirm that they were not subsequently diagnosed with KD. The current version of KDpredictor is therefore designed to distinguish high-risk KD cases from the broader population of febrile illnesses. In clinical practice, differentiating KD from KD-mimicking conditions remains a challenge, particularly in the early stages. Future work will involve sub-classifying the febrile control group as more detailed diagnostic information becomes available, enabling finer-grained analysis and potentially improving the specificity of KDpredictor.

## 5 Conclusions

In this study, we introduced KDpredictor, a tool specifically designed for objective KD risk identification based solely on features extracted from routine blood tests without reliance on clinical symptoms. KDpredictor has demonstrated high performance in identifying KD across patients recruited in different medical centers, underscoring its robustness and generalizability. In addition to its diagnostic support, the key predictive features identified by KDpredictor offer new insights that may inform future KD research. Importantly, KDpredictor is intended to support, not replace, clinical decision-making, as physician judgment remains paramount. We believe that this tool has the potential to address diagnostic challenges, particularly in regions with limited access to experienced pediatricians, and to contribute meaningfully to improving pediatric healthcare delivery.

## CRediT authorship contribution statement

**Tzu-Hsien Yang:** Writing – review & editing, Writing – original draft, Visualization, Validation, Software, Project administration, Methodology, Investigation, Formal analysis. **Ying-Hsien Huang:** Writing – original draft, Investigation, Data curation, Conceptualization. **Yuan-Han Lee:** Software, Methodology, Investigation. **Jie-Nan Lai:** Software. **Kuang-Den Chen:** Data curation, Conceptualization. **Mindy Ming-Huey Guo:** Data curation. **Yan Pan:** Data curation. **Chun-Yu Chen:** Data curation. **Wei-Sheng Wu:** Writing – review & editing, Writing – original draft, Project administration, Investigation, Formal analysis, Conceptualization. **Ho-Chang Kuo:** Writing – review & editing, Writing – original draft, Investigation, Formal analysis, Conceptualization.

## Declaration of Competing Interest

The authors declare that they have no known competing financial interests or personal relationships that could have appeared to influence the work reported in this paper.
